# The Proprotein Convertase Encoded by *amontillado* (*amon*) Is Required in *Drosophila* Corpora Cardiaca Endocrine Cells Producing the Glucose Regulatory Hormone AKH

**DOI:** 10.1371/journal.pgen.1000967

**Published:** 2010-05-27

**Authors:** Jeanne M. Rhea, Christian Wegener, Michael Bender

**Affiliations:** 1 Department of Genetics, University of Georgia, Athens, Georgia, United States of America; 2 Emmy Noether Neuropeptide Group, Department of Animal Physiology, Philipps University, Marburg, Germany; University of California San Francisco, United States of America

## Abstract

Peptide hormones are potent signaling molecules that coordinate animal physiology, behavior, and development. A key step in activation of these peptide signals is their proteolytic processing from propeptide precursors by a family of proteases, the subtilisin-like proprotein convertases (PCs). Here, we report the functional dissection of *amontillado* (*amon*), which encodes the *Drosophila* homolog of the mammalian PC2 protein, using cell-type specific inactivation and rescue experiments, and we show that *amon* is required in the islet-like adipokinetic hormone (AKH)–producing cells that regulate sugar homeostasis. In *Drosophila*, AKH acts analogously to vertebrate glucagon to increase circulating sugar levels from energy stores, while insulin-like peptides (DILPs) act to decrease sugar levels. *amon* mutant larvae have significantly reduced hemolymph sugar levels, and thus phenocopy larvae where the AKH–producing cells in the corpora cardiaca have been ablated. Reduction of *amon* expression in these cells via cell-specific RNA inactivation also results in larvae with reduced sugar levels while expression of *amon* in AKH cells in an *amon* mutant background rescues hypoglycemia. Hypoglycemia in larvae resulting from *amon* RNA inactivation in the AKH cells can be rescued by global expression of the *akh* gene. Finally, mass spectrometric profiling shows that the production of mature AKH is inhibited in *amon* mutants. Our data indicate that *amon* function in the AKH cells is necessary to maintain normal sugar homeostasis, that *amon* functions upstream of *akh*, and that loss of mature AKH is correlated with loss of *amon* activity. These observations indicate that the AKH propeptide is a proteolytic target of the *amon* proprotein convertase and provide evidence for a conserved role of PC2 in processing metabolic peptide hormones.

## Author Summary

Peptide hormones are important signaling molecules that coordinate physiology, behavior, and development. A key step in production of peptide hormones is the proteolytic cleavage of larger inactive precursors by prohormone convertases (PCs). Studies in a variety of organisms, including humans, have shown that deficiencies in PC genes lead to complex and detrimental changes. We used fruitfly genetics to dissect the function of *Drosophila* PC2, encoded by the *amon* gene, in the regulation of carbohydrate metabolism. We found that *amon* is expressed in endocrine cells of the corpora cardiaca that produce the sugar-mobilizing adipokinetic hormone (AKH), a functional analog of vertebrate glucagon. Previous studies suggest that the AKH–producing cells are homologs of the glucagon-producing islet alpha-cells in the pancreas. We found that flies with *amon* deficiency had significantly reduced hemolymph (insect “blood”) sugar levels. Using cell-type specific inactivation and rescue experiments, we show that *amon* expression in the AKH cells is necessary and sufficient for normal sugar regulation. We also demonstrate that AKH production is inhibited in *amon* mutants. Our results indicate that *amon* is necessary to maintain normal hemolymph sugar levels by activating AKH and suggest a conservation of PC2 function in processing peptide hormones between flies and mammals.

## Introduction

Most peptide hormones and neuropeptides are synthesized as part of larger inactive precursor molecules that must be enzymatically processed by the subtilisin-like proprotein convertases (PCs) to yield bioactive peptide signals. Processing of peptide and neuropeptide hormones is an important regulatory step. Many prohormone precursors encode multiple peptides with distinct functions [1]–[3] and a given precursor may be differentially processed in a cell-specific fashion depending on the PC processing enzyme expressed [4]–[6]. In some cases, the rate and extent of prohormone processing have been shown to be controlled by regulation of PC expression [5], [7], [8]. Modulation of PC expression depending on cell type or upon changing physiological conditions therefore constitutes an important regulatory input for peptide and neuropeptide hormone signaling. Finally, PC activity may be regulated by the action of serpin protease inhibitors [9]–[11], highlighting another control point for peptide hormone production.

*Drosophila* is a favorable model system for understanding how PCs function at a cellular level to regulate physiology, behavior, and development because of facile genetics, including tools that allow cell-type specific expression and inactivation. In addition, much is already known from *Drosophila* and other insect systems about the endocrine control of energy metabolism and physiology [12]–[15], neuropeptide control of behavior [16]–[19] and peptide hormone control of developmental progression [20]–[23]. The *amontillado* (*amon*) gene (CG6438, Flybase ID FBgn0023179), which encodes a homolog of mammalian PC2 [24], is one of three members of the PC family that have been identified in Drosophila [18]. The remaining two genes, *dfurin1* and *dfurin2*, encode homologs of mammalian furin [25], [26]. *amon* is expressed in neuroendocrine cells [24] and genetic studies have shown that *amon* is broadly required throughout the *Drosophila* life cycle [24], [27], [28]. Hwang et al. [29] have shown that the *amon* protein is an active protease on a KR containing synthetic peptide when expressed in S2 *Drosophila* cells. It is nevertheless unclear from these studies whether *amon* is involved in the processing of any native *Drosophila* peptide *in vivo*.

Like humans, *Drosophila* and other insects employ two antagonistically acting hormones to maintain sugar homeostasis. AKH is the insect analog of vertebrate glucagon and is known to regulate both lipid and sugar mobilization from the fat body during activities such as flight and locomotion [30]–[35] or under conditions of starvation [13], [36], [37]. In insects, trehalose is the major form of sugar found in the hemolymph along with monomeric glucose, and consists of two 1,1-conjugated glucose molecules [38]. AKH also inhibits the synthesis of RNA, fatty acids and proteins in the fat body, the insect equivalent of adipose tissue [39]. In *Drosophila*, AKH is synthesized in endocrine cells of the corpora cardiaca (CC) as a preprohormone containing a signal peptide, a single AKH of 8–10 amino acids, and a carboxyterminal peptide [40]–[42]. Before AKH is released, the mature AKH peptide is enzymatically cleaved from the carboxyterminal peptide at a dibasic processing site of the kind typically recognized by PCs [40] and then further processed by a carboxypeptidase and amidating enzymes [43]. In contrast to AKH, the *Drosophila* insulin-like peptides (DILPs) act to lower glucose levels in the hemolymph [15]. The DILPs also possess dibasic cleavage sites and are similar in structure to mammalian insulin [44]. While this suggests that PCs are involved in the processing of metabolic peptide hormones, *amon* has so far not been linked to a metabolic phenotype in flies.

Here we use cell-type specific inactivation and rescue experiments to show that *amon* function is required in the AKH cells to maintain normal sugar homeostasis. We find that hypoglycemia resulting from *amon* RNA inactivation in the AKH cells can be rescued by heat-shock driven expression of the *akh* gene, indicating that *amon* acts upstream of *akh*. In addition, production of mature AKH is inhibited in *amon* mutants as measured by mass spectrometric profiling. Together, our results are consistent with the model that the AKH propeptide is a proteolytic target of the *amon* proprotein convertase and suggest a conservation of PC2 function in the processing of peptide hormones regulating sugar homeostasis in insects and vertebrates. Our results also suggest that the *amon* inactivation and rescue reagents reported here will be generally useful, e.g. in conjunction with cell ablation experiments, to cell-specifically define the functional significance of signals produced by peptidergic cells in *Drosophila*.

## Results

### *amon* mutants have reduced hemolymph sugar levels

**Figure 1:**
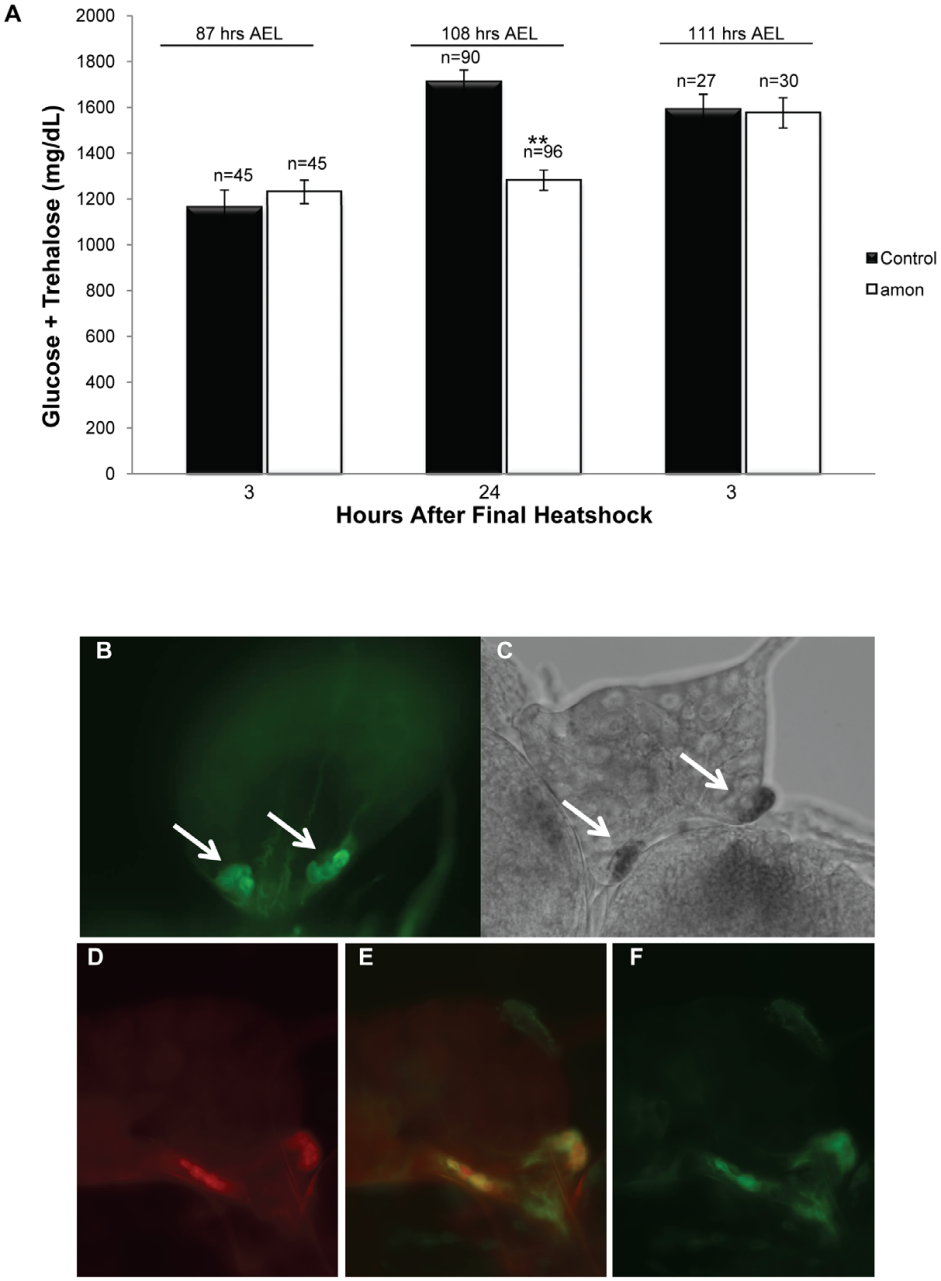
Larvae lacking functional *amon* have reduced hemolymph sugar levels. (A) Bars indicate combined glucose and trehalose hemolymph levels in control siblings (black) and *amon^Q178st^* mutants (white). Hemolymph carbohydrate levels were measured in control and *amon* mutant larvae collected 3 h (left bars) and 24 h (center bars) after the last in a series of three heatshocks (at 36, 60, and 84 h AEL) and in control and *amon* mutant larvae collected 3 h (right bars) after the last of a series of four heatshocks (at 36, 60, 84, and 108 h AEL). (B) *amon-gal4* drives expression of *uas-CD8-GFP* in the corpora cardiaca (CC) cells of the ring gland (white arrows). (C) *In situ* hybridization of an *amon* probe to the ring gland. White arrows indicate signal in the ring gland CC cells. (D)AKH cells are visualized using an α-AKH antibody. Signal from *amon-gal4* (F) co-localizes to the AKH cells (E). n = number of larvae assayed; larvae were pooled in groups of three. **p<0.0001, Students T-Test.

*amon* mutants die early in development, with most arresting as first instar larvae exhibiting molting defects [28]. In order to obtain sufficient volumes of hemolymph for sugar level determination, we provided *amon* expression via a *hs-amon* transgene to rescue mutants past the early requirements for *amon* to the third instar larval stage. While a single heatshock during the first instar larval stage was sufficient to rescue larvae to the second instar larval stage, it was not sufficient to rescue them to the third instar larval stage, suggesting that *amon* protein turnover occurs in less than 24 h. Thus, total hemolymph sugar levels of *amon* mutants were determined 24 h after the last heatshock treatment as described in the Materials and Methods section. When hemolymph was collected from *amon* mutants 3 h after the final heatshock, combined sugar levels (1230 mg/dL, SEM = 51.73) were similar to those seen in wild-type sibling controls (1163 mg/dL, SEM = 76.64, Figure 1A, left bars). Twenty-four hours after the final heatshock, however, *amon* mutants (1281 mg/dL, SEM = 44.71) were hypoglycemic compared to control larvae (1711 mg/dL, SEM = 51.20, Figure 1A, center bars), indicating that *amon* mutants failed to properly regulate hemolymph sugar concentrations.

An additional heatshock at 108 h AEL and assay 3 h later was sufficient to restore *amon* mutant sugar levels (1575 mg/dL, SEM = 65.92) to control levels (1591 mg/dL, SEM = 65.71, Figure 1A, right bars). This observation suggests that maintenance of normal sugar levels is dependent on expression of the *amon* gene. The hypoglycemia seen in *amon* mutants is similar to that seen in larvae in which the AKH producing cells have been ablated [36], [37].

To determine if *amon* is expressed in the AKH producing cells, we created an *amon-gal4* transgenic construct in which 424 bp of *amon* promoter sequence drives expression of the yeast GAL4 protein. Combination of this construct with a *uas-cd8-gfp* construct promoted expression of GFP in the CNS in a pattern similar to that seen using an *amon* antibody [27]. Using *amon-gal4*, we also saw expression of the GFP reporter in the CC portion of the ring gland (Figure 1B) where AKH is produced. Expression of the *amon* gene in these cells was verified by *in situ* hybridization using an RNA probe directed against *amon* (Figure 1C). No hybridization was seen using a control sense probe for *amon* (data not shown). Finally, we examined the expression patterns of *amon* and AKH using the *amon-gal4* construct and an AKH antibody [45]. Figure 1D–1F show that *amon* and AKH co-localized to the CC cells of the ring gland.

### *amon* expression in the AKH-producing cells is necessary and sufficient for normal sugar regulation

**Figure 2:**
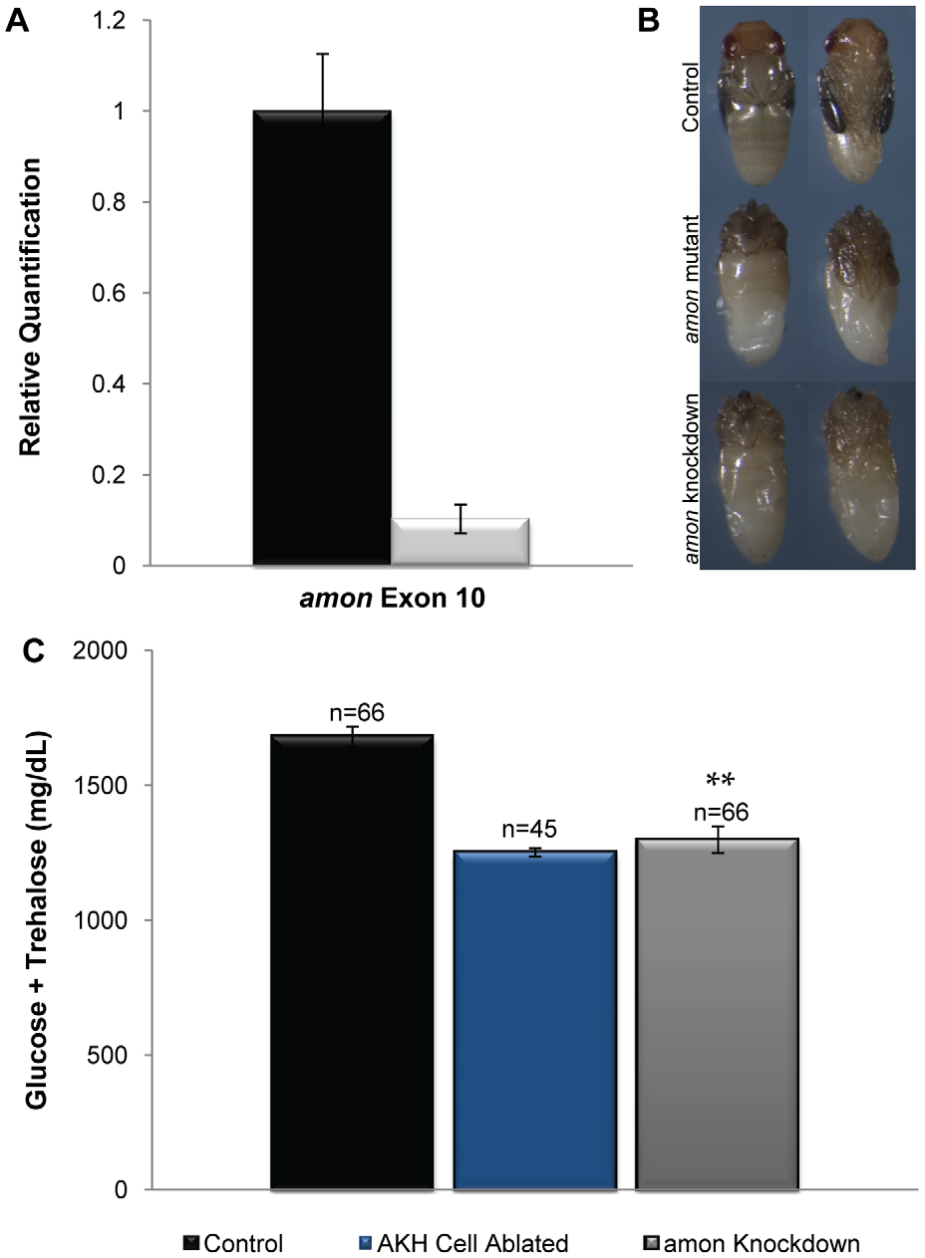
*amon* is required in the AKH producing cells for normal sugar regulation. (A) The black bar indicates *amon* transcript levels in control larvae, while the white bar indicates *amon* transcript levels when *amon-*RNAi is ubiquitously expressed. Primers specific to *amon* exon 10 were used to assess *amon* transcript levels by quantitative real time PCR. (B) Dorsal and ventral views of a control pupa (top). Middle panels represent *amon* mutants that are unable to complete metamorphosis, and die with defects in head eversion and abdominal differentiation. *amon* RNAi knockdown animals also die with phenotypes similar to *amon* mutants (bottom). (C) Combined glucose and trehalose levels of control larvae are shown in the black bar. The center blue bar shows hemolymph sugar levels in AKH ablated larvae, while the gray bar represents animals in which *amon* expression has been reduced in the AKH producing cells by RNAi. n = number of larvae assayed; larvae were pooled in groups of three. **p<0.0001, one-way ANOVA.

To ask whether *amon* expression in the AKH cells is required to maintain normal hemolymph sugar levels, we reduced *amon* expression in these cells by combining a *uas-amon*-RNAi transgene with *akh-gal4*. Figure 2A and 2B shows that ubiquitous expression of the *uas-amon-*RNAi transgene via a heatshock GAL4 construct reduced *amon* transcript levels by 90% as measured by quantitative real time PCR (Figure 2A), and that ubiquitous expression of this transgene phenocopied *amon* mutants. Ninety-one percent of *amon* knockdown animals (n = 182) died when the *uas*-*amon-*RNAi construct was expressed using *hs-gal4*; expression of the *uas-amon-*RNAi transgene using an *actin-gal4* construct resulted in complete lethality (n = 60). In addition, the phenotypes observed in knockdown animals that arrest during pupal development using the *hs-gal4* driver (Figure 2B, bottom) resembled *amon* mutants (Figure 2B, middle), including a failure to evert the head and a failure of the abdomen to differentiate.

Knockdown of *amon* activity in the AKH cells using *akh-gal4* and the *uas-amon-RNAi* transgene resulted in a significant decrease in combined glucose and trehalose levels (Figure 2C, gray bar) relative to control larvae (Figure 2C, black bar). In these experiments, this difference was similar to AKH cell ablated larvae produced by combining *akh-gal4* and a *uas-reaper* transgene (Figure 2C, blue bar). Thus, *amon* activity is necessary in the AKH producing cells to maintain normal sugar homeostasis.

**Figure 3:**
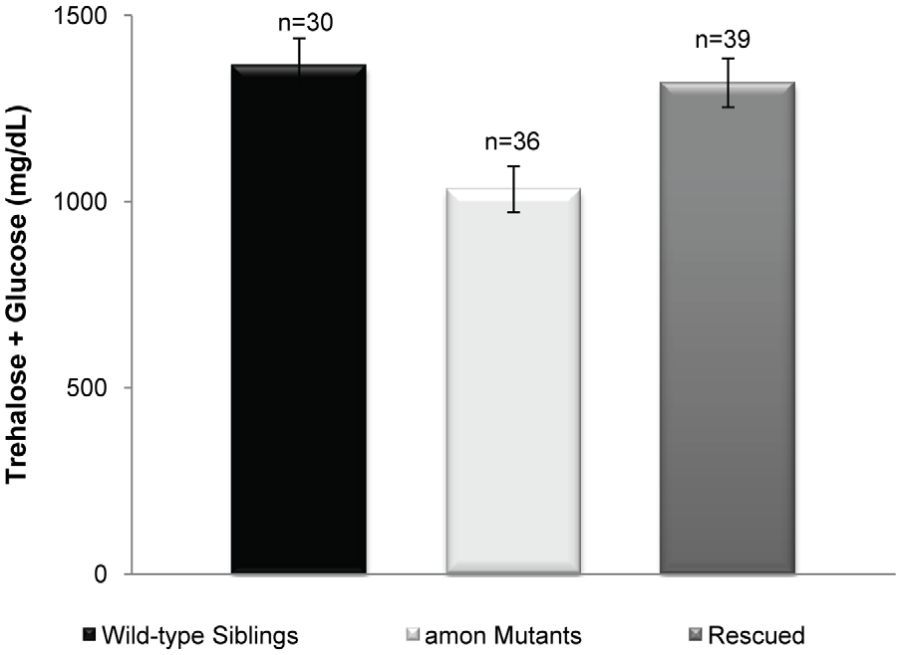
Expression of *amon* in the AKH cells of an *amon^C241Y^* mutant is sufficient to rescue hypoglycemia. The gray bar represents larvae in which *amon* expression has been restored in the AKH producing cells (*yw; uas-amon/hs-amon; Df(3R) Tl-X e/akh-gal4, amon^C241Y^*) as compared to *amon* mutants (*yw; uas-amon/hs-amon; Df(3R) Tl-X e/amon^C241Y^* white bar) and control siblings (*yw; uas-amon/hs-amon; Df(3R) Tl-X e* or *amon^C241Y^/TM3 Sb Ser y+ e,* black bar). n = number of larvae assayed; larvae were pooled in groups of three. p<0.0015, one way ANOVA.

To ask whether *amon* activity in the AKH producing cells is sufficient to maintain hemolymph sugar concentrations, we expressed *amon* in the AKH cells in an *amon^C241Y^* mutant background. Expression of *amon* in the AKH cells was achieved by combining a *uas-amon* construct and the *akh-gal4* driver in an *amon* mutant background. Expression of *amon* in these cells alone was not sufficient to rescue the early developmental requirements for *amon*. Therefore, in order to obtain larvae large enough for sugar determination, we combined the *hs-amon* construct with the *uas-amon* and *akh-gal4* constructs in an *amon* mutant background. *amon* mutants were rescued by expressing *amon* via the heatshock promoter once every 24 h until the third instar larval stage. In this background, *amon* is expressed in the AKH cells by virtue of the *uas-amon* and *akh-gal4* constructs, allowing us to examine requirements for *amon* in sugar homeostasis. Sugar levels were assayed approximately 24 h after the final heatshock as described earlier. Expression of *amon via* a *uas-amon* transgene in the AKH cells of an *amon* mutant resulted in larvae with wild-type sugar levels (Figure 3, black versus gray bar). This observation indicates that restoring *amon* activity in the AKH producing cells in an *amon* mutant background is sufficient to rescue the hypoglycemic defect.

### Ubiquitous expression of AKH rescues hypoglycemia induced by *amon* knockdown in AKH-producing cells

**Figure 4:**
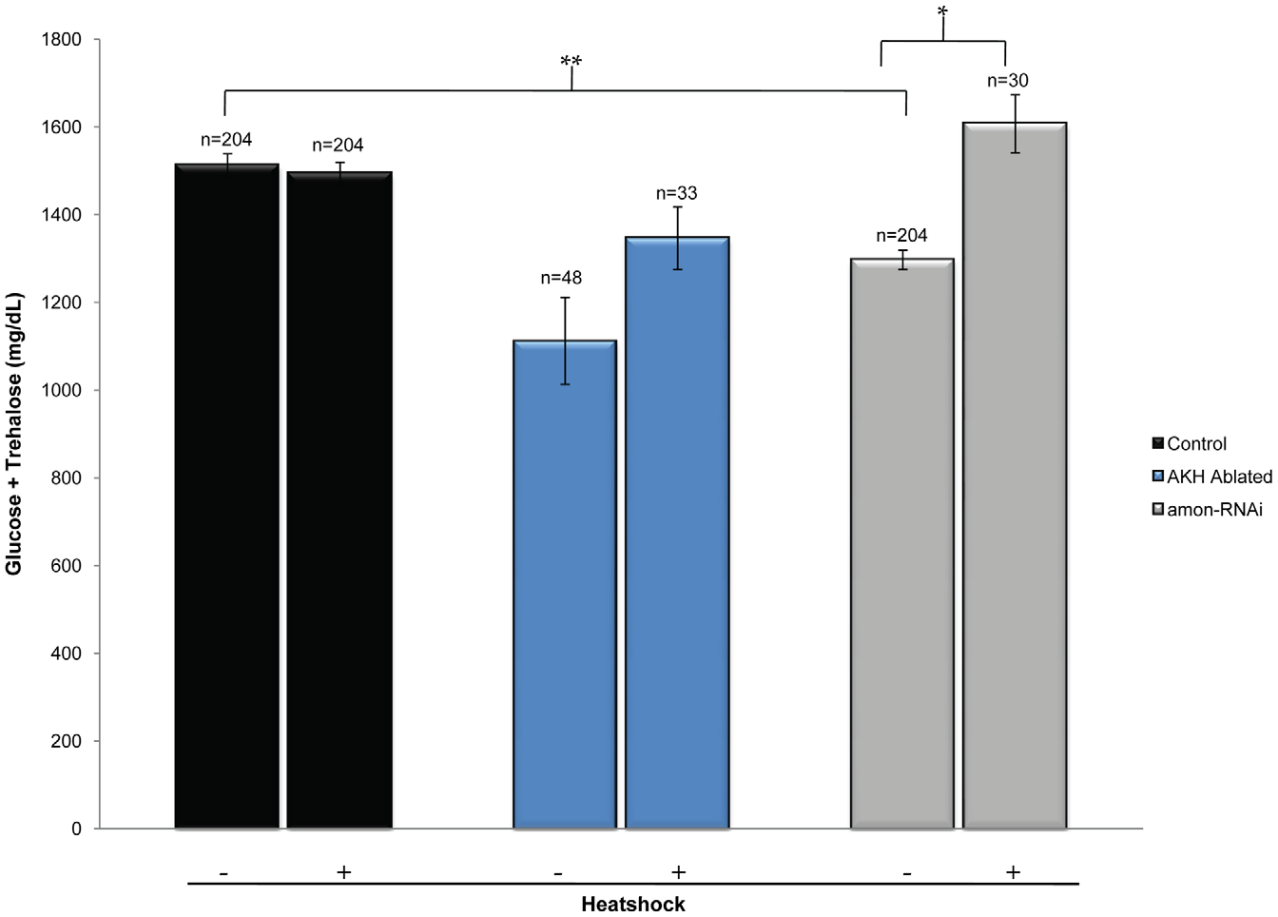
Ubiquitous expression of AKH rescues the hypoglycemic defect seen in *amon* knockdown larvae. The left black bar represents wild-type levels of combined glucose and trehalose (*yw; hs-akh/+; akh-gal4/+*). The left blue bar represents combined sugar levels of AKH ablated larvae (*yw; hs-akh/uas-reaper; akh-gal4/+*) while the left gray bar shows glucose and trehalose levels in which *amon* has been reduced in the AKH cells by RNAi (*yw; hs-akh/uas-amon-RNAi^28b^; akh-gal4*). Bars denoted with a ‘+’ below the graph indicate combined glucose and trehalose levels following heatshock induced expression of *akh via* a *hs-akh* transgene. n = number of larvae assayed; larvae were pooled in groups of three. *p = 0.002, **p<0.0001, one-way ANOVA.

To ask whether the defect in sugar homeostasis observed in larvae in which *amon* expression has been reduced in the AKH cells *via* RNAi can be attributed to a lack of mature AKH, we expressed AKH in these larvae using the *hs-akh* transgene. It has been previously shown that the hypoglycemia induced by ablation of the AKH cells can be rescued through the expression of the *akh* gene throughout the larva using a *hs-akh* transgene [13]. Since these animals lack detectable AKH cells, this observation suggests that other cell types possess the proteolytic machinery to produce functional AKH. We found that ubiquitous expression of *hs-akh* in control larvae (*yw; hs-akh/+; akh-gal4/+)* had no effect on sugar levels, and that heatshock treatment also did not affect hemolymph sugar levels (Figure 4, black bars). In addition, we recapitulated the rescue of hemolymph sugar levels in AKH cell ablated larvae (*yw; hs-akh/uas-reaper; akh-gal4/+,* Figure 4, blue bars) demonstrated in earlier studies [13]. Finally, we show that *hs-akh* expression in larvae with reduced *amon* function in the AKH cells (*yw; hs-akh/uas-amon-RNAi^28b^; akh-gal4/+*) was sufficient to restore glucose and trehalose to control levels (Figure 4, gray bar), indicating that *amon* functions upstream of AKH to prevent hypoglycemia.

### Direct peptide profiling shows that *amon* mutants lack mature AKH peptide

**Figure 5:**
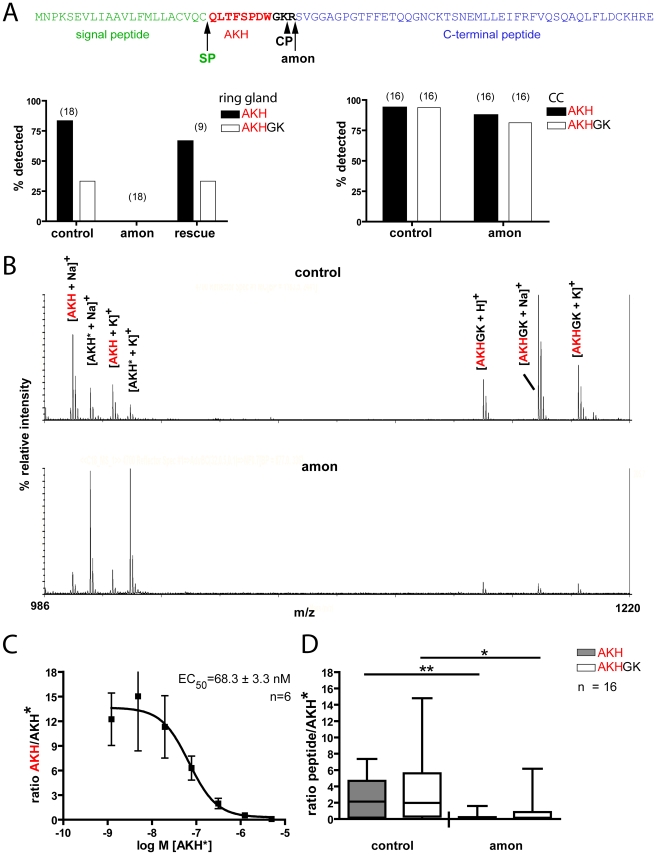
Direct peptide profiling of AKH and AKHGK in control and *amon^C241Y^* flies. (A) Model of the processing of the AKH prepropeptide (top) and profiling of the larval ring gland (left) and adult corpora cardiaca (right). AKH is processed by a concerted action of a signal peptidase (SP) and *amon*, likely followed by a two-step carboxypeptidase (CP) action that first removes the C-terminal R yielding the intermediate AKHGK. AKHG is than amidated to bioactive AKH (not shown). While AKH and AKHGK were detected in most preparations from control and rescued (continued heatshock once a day) flies, they were not detectable in *amon* larvae. (B) Original direct mass profiles from corpora cardiaca of adult control (above) and *amon* (below) flies. AKH only occurs as the characteristic [M+Na]^+^ and [M+K]^+^ adducts, whereas AKHGK also occurs as [M+H]^+^. In the control fly, both peptides show higher signal intensities as the stable isotope-labelled standard peptide (AKH*). In the *amon* fly, the signal intensity is clearly higher for AKH* than for the native peptides. As previously reported [41], [42], no other mass peaks occur in the range 990-1220 Da in direct mass spectrometric CC profiles. (C) Standard curve for adult corpora cardiaca obtained with a dilution series of AKH* added to the matrix salt, male OrR wild-type flies. The y axis shows the signal intensity ratio of native AKH/AKH*. Error bars are S.E.M. The relationship of AKH/AKH* is linear for AKH* concentrations of 50–500 nM. (D) Peptide quantification with the labeled AKH* standard at 400 nM. The concentrations of both AKH and AKHGK are significantly reduced in *amon* flies vs. controls five days after eclosion and final heatshock. *p<0.05, **p<0.01, Mann-Whitney.

The results above suggest that *amon* is responsible for the proteolytic activation of *Drosophila* AKH. To determine whether *amon^C241Y^* mutants are indeed defective in AKH processing, we directly profiled larval ring glands (a fusion product of the larval CC, corpora allata and prothoracic gland) and adult CC by MALDI-TOF mass spectrometry and compared mass signals for AKH and processing intermediates between wild-type and *amon* mutant flies. Earlier work has shown that AKH and a processing intermediate with the C-terminal extension GK (AKHGK, Figure 5A) appear as dominant mass signals in direct MALDI-TOF mass spectrometric profiles of single larval ring glands or adult CC [41], [42].

We detected mature AKH and AKHGK in 89% of third instar larval ring glands from control flies, but not in any *amon* mutant ring gland (n = 18, Figure 5A, left graph). A continuous heatshock expression of *amon* rescued larval AKH and AKHGK production to control levels (Figure 5A left), indicating that *amon* is required for the proteolytic cleavage of AKH. Likewise, a heatshock 1 d before dissection after a 2 d heatshock break rescued AKH production in larvae (100% AKH/80% AKHGK detections, n = 5). To test whether *amon* similarily affects AKH and AKHGK production in adult flies, we profiled the CC/hypocerebral ganglion complex of 5 d old adult flies heatshocked until eclosion. In both control (94%) and *amon* mutant (88%, n = 16) adults, mass peaks corresponding to AKH and AKHGK were detected (Figure 5A right), typically with a decreased signal-to-noise ratio for the AKH and AKHGK peaks in *amon* mutants. While this decreased signal-to-noise ratio indicated a lower amount of AKH and AKHGK in *amon* mutant CC, it is problematic to use MALDI-TOF signals *per se* to quantify peptides mainly due to non-homogenous analyte distribution in the co-crystallite and ion suppression effects (see [46], [47]). A solution to minimize these adverse effect is a proper choice of matrix, decomplexing of the sample and the application of chemically similar internal standards (see [46], [47]). We used α-cyano-4-hydroxycinnamic acid as a matrix since it results in relatively homogenous signals and has been found suitable for quantitative analysis of peptides by MALDI-TOF MS [47]. In contrast to homogenized samples, the on-plate extraction during the direct tissue profiling allows only small peptides to permeate in larger amounts through the cell membrane [42], which favourably reduces sample complexity. For quantification, we added a constant amount of heavy isotope-labeled AKH* as chemically identical internal standard with the matrix, and calculated the ratio of the relative signal intensity of native AKH or AKHGK vs. AKH*. A standard curve obtained from CC of 1d old OrR flies showed that the ratio of the relative intensities of native AKH and AKH* was linear when AKH* is present in a concentration of 50–500 nM (Figure 5C). With the matrix, we therefore added 400 nM AKH* as internal standard throughout the quantitative measurements. Control flies 5 days after eclosion and last heat shock showed a significantly higher ratio of AKH/AKH* than *amon* mutant flies (median: 2.14 vs. 0.13 (n = 15/16), Mann-Whitney test, Figure 5D). They also showed a significantly higher ratio of AKHGK/AKH* (median 1.974 vs. 0.15 (n = 16) Mann-Whitney test, Figure 5D).

The results above imply that the production of AKH is impaired in both larval and adult *amon* mutants, while the depletion of AKH after stopping heatshock-induced expression of *amon* happens slower in adult than in larval flies. To test for a slow depletion in adult flies, we profiled the CC of males and females 1 and 10–14 d after eclosion and last heatshock. At 1 d after eclosion and last heatshock, the AKH ratio between control and *amon* flies (median: males: 0.64 vs. 0.26, females: 0.18 vs. 0.14, ratio±s.e.m. (n = 8–10)) did not differ significantly (p>0,05, Mann-Whitney test,). The ratios became, however, significantly different at d10–14 (median: males: 1.05 vs. 0.03, females: 1.99 vs. 0.04 (n = 6–8), p<0.01, Mann-Whitney test). Based on this ratio data and the assumption of a unity slope of the AKH/AKH* ratio, we calculated the mean amount of AKH present in the CC (Table 1). The AKH levels one day after eclosion and last heat shock ranged between 20–40 fmol/CC and did not significantly differ between control and *amon* flies. On day 5, AKH levels had increased by more than 4fold in control but not in *amon* flies. At day 10–14, AKH levels were similar to day 5 in control flies, while *amon* flies showed reduced levels compared to day 1 and 5. Significant sex-specific differences in AKH levels were not apparent.

**Table 1:** Calculated levels of AKH in the corpora cardiaca of individual flies.

Age*	Males		Females	
	Control	amon	Control	amon
1	38±8 (8)	20±7 (8)	21±7 (9)	22±8 (10)
5	174±49 (3)	20±8 (3)	178±54 (13)	22±10 (12)
10–14	167±83 (8)	11±7 (7)	205±114 (7)	6±3 (6)

*Days post eclosion and last heatshock.

(mean ± sem in fmol, (n)).

## Discussion

The *Drosophila* proprotein convertase *amon* is a homolog of mammalian PC2 and is expressed in numerous peptidergic neurons [24], [27]. Phenotypic analysis of animals lacking functional *amon* have revealed a requirement for this gene during embryonic hatching [24], molting [28], and metamorphosis [27], [28]. In addition, the *amon* protein is an active protease on a KR containing synthetic protein when expressed in *Drosophila* S2 cells [29]. While these observations suggest that *amon* may be required for the proteolytic activation of one or more secretory peptides, it has not been shown that flies lacking *amon* indeed show a deficiency in peptide production, nor have any of the observed phenotypes of *amon*-deficient flies been linked to a particular peptide or peptidergic cell. In this study, we identify AKH as a proteolytic target of *amon* activity, and show that flies lacking *amon* show a severe deficiency in AKH production. We link this deficiency to a new *amon* phenotype (hypoglycemia) and show that *amon* activity is both necessary and sufficient in the AKH cells to maintain sugar homeostasis.

### A conserved role of PC2 in metabolic hormone processing

A similar requirement for PC2 activity in the maintenance of sugar homeostasis as described here for *Drosophila* has also been reported in mice [4], [48]. While in vertebrates PC2 is expressed in the glucagon-producing pancreatic alpha-cells [4], [48], we demonstrate that *amon* co-localizes with AKH in the endocrine corpora cardiaca cells (Figure 1). These endocrine cells have been suggested to be homologs of the pancreatic alpha-cells [49] and use glucose-sensing and response mechanisms similar to islet cells [13]. We further report that *amon* mutants fail to produce bioactive AKH, a phenotype similar to PC2 null mice that show defects in the processing of several peptide precursors including proglucagon in the pancreatic alpha-cells [50], [51], prosomatostatin, and proopiomelanocortin [52], [53] (Figure 5). These data not only support our model that AKH production is regulated by *amon* activity, they also suggest an evolutionarily conserved function of PC2 in the processing of metabolic hormones. This view is consistent with the sequence conservation of *amon* and vertebrate PC2 and with the small number of PCs expressed in the *Drosophila* and mammalian endocrine system.

### Peptide quantification by direct mass-spectrometric profiling

Direct MALDI-TOF mass spectrometric profiling holds several advantages over liquid chromatography/mass spectrometry (LC/MS) analyses. Most important, it is quick and can be performed on tissues from single animals. Direct MALDI-TOF mass spectrometric peptide quantification has been performed on homogenates of vertebrate and invertebrate endocrine and nervous tissue [54], [55]. We modified this approach by on-plate extraction of isolated CC which is more time-efficient and further minimizes analyte loss, and found a linear signal relationship between analyte and standard around the 100 nM range with the use of an internal stable isotope-labeled standard. This suggests that the method can be used quantitatively within this concentration range. We did, however, not establish calibration curves or precision rates for *amon* or control flies on the same plate and under the same conditions, which is highly laborious with *amon* mutants. Our assumption of a unity slope of the ratio curve underlying the calculations of AKH levels may thus not be correct. Nevertheless, our estimates of the AKH levels in the CC of 5 and 10–14 day old adult control flies (around 170–200 fmol/CC) are surprisingly close to the AKH levels previously determined by HPLC from thorax extracts containing the CC (around 100–140 fmol/fly) [32], [40]. In contrast to the results obtained by HPLC, we could not find a significant sex difference in the AKH levels, and observed a lower AKH content in freshly ecdysed flies. While the HPLC-quantification needed extracts from 50 flies, our data originates from single tissue measurements. Our results suggest quantitative direct peptide profiling as a suitable and time-efficient method to (semi)quantify peptide hormones in flies and other small animals which contain small amounts of peptides and are therefore not easily tractable by conventional quantification methods such as enzyme assays or HPLC.

### AKH turnover differs between larval and adult flies

In third instar larvae, AKH was undetectable 3 days after the last heat shock, while it was still found in adult flies that had received their last heat shock 5 or more days ago. This suggests that AKH turnover is considerably higher in larvae than in adults under laboratory conditions. Whether this is due to differences in release or production rates, or the size of the AKH storage pool, has yet to be determined. In nature, where longer flights or periods of carbohydrate shortage occur, adult AKH turnover may be much higher. The increase of the AKH level between day 1 and 5 in control flies suggests -similar to the situation in locusts [56] - that AKH production is not coupled to release. Rather, processed AKH seems to be stored in large reserve pools. Interestingly, the biosynthesis of pancreatic glucagon similarly seems not to be coordinated with glucagon release, since e.g. arginine and palmitate induce increased glucagon release, but do not increase glucagon mRNA levels in isolated rat pancreatic islets [50], [57], [58].

### Genetic tools to study peptide processing, release, and function in a cell-specific manner

The genetic tools described here allow a reduction of *amon* activity and the restoration of *amon* expression in a highly cell-specific fashion in an *amon* mutant background. These potentially very powerful tools can now be used to identify cell-type specific requirements of *amon* and to trace the observed phenotypes of *amon* deficient flies to identified peptidergic signaling networks, revealing their functions. In addition, the apparent evolutionarily conserved mechanism of peptide hormone production by the proprotein convertase family presents the opportunity to use *Drosophila* and the described genetic tools to study the regulatory mechanisms behind peptide hormone synthesis, processing, and release and to apply the knowledge to mammals.

For example, the hypoglycemic defect of PC2 null mice can be rescued by providing a constant supply of glucagon *via* an osmotic pump [59]. While this result indicates that supply of glucagon is sufficient to correct the misregulation of glucose levels in PC2 null mice, it does not provide a direct correlation between loss of PC2 activity in the glucagon producing cells with decreases in blood glucose levels. Thus, a more powerful approach would be to reduce PC2 expression only in the alpha-cells of the pancreas and then determine the effect on glucose levels. In *Drosophila*, such a cell specific reduction of *amon* can be easily achieved by expression of *uas-RNAi* constructs using tissue-specific Gal4 expression constructs. Subsequent replacement of hormones can then be accomplished either through direct injection of synthetic hormones, or through broad expression of the cDNA fused to a heatshock promoter. Since peptide processing requires a dedicated enzyme machinery (PCs, carboxypeptidases, amidating enzymes), it is likely that the heatshock-induced expression of AKH or other peptides leads to properly processed and bioactive peptides only in peptidergic neurons and endocrine cells [60].

In a complementary approach, *amon* can be specifically expressed in target cells in an *amon*-deficient background. Here, we have validated this approach showing not only that *amon* is required in the AKH cells to maintain glucose homeostasis in *Drosophila* (Figure 3), but also that the hypoglycemic defect can be directly attributed to a loss of AKH specifically from these cells (Figure 4).

The fly lines generated here also allow examination of PC requirements in a wide variety of cell types in a relatively short period of time. Furthermore, a combination of the genetic tools and semiquantitative direct peptide profiling as presented here has great potential for the molecular analysis of peptide processing in authentic endocrine cells and peptidergic neurons. Performing such studies in *Drosophila* is likely to provide valuable insight into the general requirements for PC function in regulating processes including growth, behavior, development, metabolism, and disease.

## Materials and Methods

### Generation of transgenic fly strains

Injections of transgenic constructs into *w1118* embryos were carried out by Duke University Model Systems Genomics. Forward (5′-ATCCAACGCAGCTGAGCAGC-3′) and reverse (5′-CGGAAGGAAAGCACAACAAG-3′) PCR primers were used to amplify an *amon* fragment extending from position -331 to +133. This fragment was cloned into the *BamHI* site of pCaSpeR4-Gal4. The homozygous viable *amon-gal491D* line was used in this study. To create the *uas-amon* RNAi construct, forward (5′-TGGCGTTGCTTATGACAG-3′) and reverse (5′-ATGTCCCGCCAAGTCAGC3′) primers for the sense insert, and forward (5′-TGGCGTTGCTTATGACAG-3′) and reverse (5′-ATGTCCCGCCAAGTCAGC-3′) primers for the antisense insert were used to amplify exons 7 and 8 of the *amon* transcript. *amon* antisense products were cloned into the puasTi vector (Amin Ghabrial, Krasnow Lab, Stanford University, Stanford, Ca) as *KpnI/XbaI* fragments. *amon* sense products were cloned into the vector as *BglII/XhoI* fragments. The homozygous viable *uas-amon-RNAi28b* line was used in this study. The *uas-amon* construct was created by digesting the *amon* cDNA sequence out of the *amon* #5–8 vector [24] as a *EcoRI/EcoRV* fragment and subcloning it into pBluescript. pBluescript-*amon* was then digested with *EcoRI* and *XhoI* and cloned into puasT [61]. The homozygous viable *uas-amon40L* line was used in this study.

### Expression of *amon-gal4* and *in situ* hybridization

*amon-gal4* was used to drive expression of GFP in cell membranes using the *uas-CD8-GFP* reporter. Ring glands were dissected from third instar larvae at approximately 108 h AEL, and mounted in glycerol. Tissues were visualized using a Hamamatsu Orca-ER digital camera (model #C4742-80). The generation of DIG-labeled *amon* probes and *in situ* hybridization to third instar larval ring glands was performed as described in [62].

### Immunohistochemistry

*yw; uas-CD8-gfp* females were crossed to *yw; amon-gal4^91D^* males. Immunostaining was carried out essentially as described [27]. For co-localization experiments, tissues were incubated with AKH antiserum (1∶1000, gift from M. Brown, University of Georgia) and anti-Green Fluorescent Protein antibody (1∶1000, Molecular Probes, Invitrogen) overnight at 4°C. Secondary antibodies used were Alexa Fluor 568 goat anti-Rabbit (1∶1000) and Alexa Fluor 488 goat anti-mouse (1∶1000).

### Heatshock rescue and trehalose assay of *amon* mutants

To obtain sufficient volumes of hemolymph, larvae were rescued to third instar larval stages by periodic heatshock driven *amon* expression. *yw; +; amon^Q178ST^/TM3 Sb Ser y+ e* virgin females were crossed to *yw; hs-amon; Df(3R)Tl-X e/TM3 Sb Ser y+ e* males and placed in an egg collection chamber containing a grape juice agar plate with fresh yeast paste at 25°C. Four hour egg collections were used. Beginning at 36 h AEL, plates were heatshocked at 37°C for 30 min. Subsequent heatshocks were performed at 60 h, 84 h, and in one assay at 108 h AEL. At either 87 h, 108 h, or 111 h AEL, trehalose and sugar measurements were done using pooled hemolymph from groups of three larvae as previously described [15]. *Canton S* larvae were heat-shocked and assayed in the same manner as the *amon* mutants described above. To assay combined glucose and trehalose levels of larvae in which *amon* function was removed in the AKH producing cells, *yw; +; akh-gal4* virgin females were crossed to either control males (*w^1118^*) or experimental males (*w; uas-amon-RNAi^28b^; +*). As an additional control, *yw; +; akh-gal4* virgin females were crossed to *w; uas-reaper; +* males. Flies were raised on standard fly food and incubated at 25°C. At 108 h AEL, glucose and trehalose measurements were performed as described above using feeding third instar larvae. To determine if *amon* functions upstream of AKH in controlling sugar homeostasis, *yw; hs-akh; akh-gal4* virgin females were crossed to either control male flies (*w^1118^*) or experimental male flies (*yw; uas-amon-RNAi^28b^; +)*. As an additional control, *yw; hs-akh; akh-gal4* virgin females were crossed to *w; uas-reaper; +* males. Crosses and egg collections were performed as described above. At 36 h AEL, approximately 100 first instar larvae from control and experimental crosses were transferred to standard fly food. Beginning at 36 h AEL, larvae were heatshocked at 37°C for 45 min every 8 h. At 108 h AEL, glucose and trehalose measurements were performed as described. To determine if *amon* function in the AKH producing cells is sufficient to maintain sugar homeostasis, *yw; uas-amon; Df(3R) Tl-X e/TM3 Sb Ser y+ e* virgin females were crossed to *yw; hs-amon; akh-gal4, amon^C241Y^/TM3 Sb Ser y+ e* males. As a control cross, *yw; uas-amon; Df (3R)Tl-X e/TM3 Sb Ser y+ e* virgin females were crossed to approximately *yw; hs-amon; amon^C241Y^/TM3 Sb Ser y+ e* males. Crosses and egg collections were performed as described above. At 36 h AEL, plates were heatshocked at 37°C for 30 min. Subsequent heatshocks were performed at 60 h and 84 h AEL. At 108 h AEL trehalose and glucose measurements were done as described previously.

### Quantitative real-time PCR

*yw; uas-amon-RNAi^28b^* virgin females were crossed to *hs-gal4* males in an egg collection chamber containing an apple juice plate with fresh yeast paste, and maintained at 27°C. As a control, *w^1118^* virgin females were crossed to *hs-gal4* males in the same manner. After 48 h, a 4 h egg collection was taken. Beginning at 36 h AEL, egg collection plates were heatshocked every 12 h for 1 h at 37°C. Total RNA was isolated from 10 whole third instar larvae at 108 h AEL with TRIzol reagent (Invitrogen), treated with DNaseI, and 5 µg were used for reverse transcription using the Transcriptior First Strand cDNA Synthesis Kit (Roche). The *amon* transcript was quantified using an Applied Biosystems 7500 Real-Time PCR system according to the manufacturer's instructions. Two sets of primers were used, targeting either exon 10 or exon 11: exon 10 forward (5′-GCCGGCGCCATGGT-3′) and reverse (5′-ATAGCGCGGTGGCACTGA-3′), and exon 11 forward (5′-TTCAACTCGCCCCAAACAC-3′) and reverse (5′-ATGCAGGACCAAGGACCATTC-3′). Ribosomal protein 49 (rp49) was used as an endogenous control.

### Lethal phase analysis of *amon-RNAi* animals

Crosses and egg collections were performed as described in Quantitative Real Time PCR. Beginning at 36 h AEL, both control and experimental animals were heatshocked at 37°C for 1 h every 12 h until approximately 12 days (d) AEL. Death was scored once every 24 h. Pictures were taken with a digital camera (Hamamatsu 3CCD) mounted to a Leitz dissecting scope.

### Quantitation of growth defects

*yw; uas-amon; Df(3R)Tl-X e/TM3 Sb y+ e* virgin females were mated to either control males (*yw; +; amon^C241Y^ e/TM3 Sb Ser y+ e)* or experimental males (*yw; +; akh-gal4, amon^C241Y^ e/TM3 Sb y+ e*) and were placed in egg collection chambers at 25°C. Four hour egg collections were taken. Larvae were sorted at 36 h AEL into mutant or control classes using the *yellow* marker. *amon* mutant larvae (*yw; uas-amon/+; amon^C241Y^ e/Df(3R)Tl-X e*), rescued larvae (*yw; uas-amon/+; akh-gal4, amon^C241Y^ e/Df(3R)Tl-X e)* and control larvae (*yw; uas-amon; amon^C241Y^ or Df(3R)Tl-X e/Tm3 Sb Ser y+ e)* were washed and killed by microwave as described [15]. Length measurements were made from photographs taken with a digital camera (Hamamatsu 3CCD) mounted to a Leitz dissecting scope.

### MALDI-TOF mass spectrometric profiling

Eggs of *yw; +; amon^C241Y^/TM3 Sb Ser y+ e* X *yw; hs-amon; Df(3R)Tl-X e/TM3 Sb Ser y+ e* flies were collected every morning, and heatshocked every 24 h until the larvae had reached the third instar. Three days later, the CNS was dissected free from surrounding tissue in standard *Drosophila* saline. For adult flies, heatshock was continued until eclosion and then stopped, and the corpora cardiaca (CC)/hypocerebral ganglion were dissected from 5 d old adult males as described [63]. Larval ring glands or the adult CC/hypocerebral ganglion were punched out with pulled glass capillaries, spottet directly onto the MALDI target and left to dry. For the ring gland, matrix (saturated solution of recrystallized α-cyano-4-hydroxycinnamic acid in MeOH/EtOH/Aq.bidest 30/30/40%) was added in small nanoliter volumes with a manual oocyte injector (Drummond Digital, Broomall, PA, USA). For adult CC, 200 nl of matrix was added to each sample with a micropipette. For peptide quantifications, 400 nM heavy isotope-labeled AKH* (pGlu-Leu[^13^C6, ^15^N]-Thr-Phe-Ser-Pro-Asp-Trp-amide, Mw = 982.5 Da, Iris Biotech, Marktredwitz, Germany) were added beforehand to the matrix solution. Low protein-binding plasticware was used throughout to minimize peptide loss. MALDI-TOF mass spectra were acquired in positive ion reflectron mode and delayed extraction on an Applied Biosystems Voyager DE RP MALDI-TOF or 4800+ MALDI TOF/TOF mass spectrometer (for quantifications). To suppress matrix ions, the low mass gate was set to 850 Da, with a focus mass of 1100 Da. For quantification, laser power was first adjusted with one sample to provide optimal signal-to-noise ratios, and then kept constant for all samples on the MALDI target. Each spectrum consisted of five subspectra with 50 shots each. For standard curves, adult OrR flies 1 d after eclosion were used; larvae were not sexed. For each *amon* fly, we measured a control fly taken from the same bottle that had either eclosed on the same day (adults) or originated from the same day of egg laying (larvae) to minimize possible age, food or population density effects. Data were analyzed with Data Explorer 4.3 software (Applied Biosystems). For quantification, mass spectra were base-line corrected and de-isotoped, and the relative peak intensities for the different adducts ([M+H]^+^,[M+Na]^+^,[M+K]^+^) of AKH and AKHGK were summed. Finally, the ratio of the resulting relative peak intensities of AKH/AKH* and AKHGK/AKH* was calculated. Statistics were performed using GraphStat Prism 4.0 (GraphStat Software, San Diego, CA).
